# Pancreatic Associated Manifestations in Pediatric Inflammatory Bowel Diseases

**DOI:** 10.3390/genes12091372

**Published:** 2021-08-31

**Authors:** Ugo Cucinotta, Claudio Romano, Valeria Dipasquale

**Affiliations:** Pediatric Gastroenterology and Cystic Fibrosis Unit, Department of Human Pathology in Adulthood and Childhood “G. Barresi”, University of Messina, 98124 Messina, Italy; ugocucinotta@gmail.com (U.C.); dipasquale.valeria@libero.it (V.D.)

**Keywords:** inflammatory bowel diseases, children, acute and chronic pancreatitis, pancreatic hyperenzymemia, pancreatic abnormalities

## Abstract

Inflammatory bowel diseases (IBDs) are chronic relapsing inflammatory conditions of the gastrointestinal tract, encompassing Crohn’s disease (CD), ulcerative colitis (UC) and inflammatory bowel disease unclassified (IBD-U). They are currently considered as systemic disorders determined by a set of genetic predispositions, individual susceptibility and environmental triggers, potentially able to involve other organs and systems than the gastrointestinal tract. A large number of patients experiences one or more extraintestinal manifestations (EIMs), whose sites affected are mostly represented by the joints, skin, bones, liver, eyes, and pancreas. Pancreatic abnormalities are not uncommon and are often underestimated, encompassing acute and chronic pancreatitis, autoimmune pancreatitis, exocrine pancreatic insufficiency and asymptomatic elevation of pancreatic enzymes. In most cases they are the result of environmental triggers. However, several genetic polymorphisms may play a role as precipitating factors or contributing to a more severe course. The aim of this paper is to provide an updated overview on the available evidence concerning the etiology, pathogenesis and clinical presentation of pancreatic diseases in IBD pediatric patients.

## 1. Introduction

Inflammatory bowel diseases (IBDs), encompassing Crohn’s disease (CD), ulcerative colitis (UC) and inflammatory bowel disease unclassified (IBD-U), are life-long and relapsing diseases of the gastrointestinal tract occurring in genetically predisposed individuals [1]. IBDs are currently considered as multi-systemic conditions with a bowel involvement, but wherein chronic inflammation may potentially affect other organs and systems [2,3]. In the clinical setting, it is mandatory to distinguish between true extraintestinal manifestations (EIMs) that are the result of inflammatory or autoimmune phenomena, and extraintestinal complications, due to metabolic disorders (growth failure, anemia, osteoporosis) or to drugs used for the management of the disease [4,5]. Depending on type and inter-individual variability, EIMs may have a mild and transient course or could be disabling for the patients, contributing to a higher morbidity and reduced quality of life [6].The most common sites affected are joints (axial and/or peripheral arthritis), skin (erythema nodosum, pyoderma gangrenosum, or aphthous stomatitis), eyes (episcleritis and uveitis), hepatobiliary system and pancreas [5,6,7]. Increasing evidence links IBD and pancreatic inflammation in terms of genetic susceptibility, microflora alteration and immunological features [1,2,3,4,5]. Among the broad spectrum of EIMs, pancreatic involvement is considered rare (0.7–1.6% of all EIMs), but anyhow more frequent than in the general population [8], since the IBD patients present an increased risk of pancreatic abnormalities over time. They include a heterogeneous group of entities that can occur prior, together with or after the onset of IBD, but data on actual incidence in children are lacking [7,9].

Acute pancreatitis (AP) is currently considered one of the most common pancreatic lesions in IBD [8,9], although in the pediatric setting several cases are clinically silent and the disease course is usually mild [9,10,11]. The etiology of AP in the IBD population mainly includes drugs used for the treatment of the disease and biliary lithiasis. Besides them, some forms of AP are believed to share with IBD the same pathogenesis or to be a direct consequence of the systemic inflammation and are therefore considered true EIMs of IBD [12].

A newly recognized condition in pediatrics is represented by the autoimmune pancreatitis, of which two distinct forms (Type 1 and Type 2) are described in adults [13]. Pediatric AIP has shown a distinct presentation with features similar to type 2 AIP in adults and has been associated with IBDs and other autoimmune diseases in up to 30% of cases [14].

The occurrence of asymptomatic hyperenzymemia in patients with IBD is also quite common, despite its clinical significance remains not clear [9,10,11]. Several hypotheses for asymptomatic elevation of pancreatic enzymes in IBD have been formulated, including immune abnormalities, microcirculation disorders, malnutrition and abnormal absorption of pancreatic amylase/lipase from the inflamed gut [9].

Other pancreatic manifestations, such as chronic pancreatitis, are extremely infrequent in IBD pediatric patients, and only sporadic cases have been described so far, usually diagnosed after recurrent episodes of AP [11]. The aim of this paper is to provide an updated overview on the available evidence about etiology, pathogenesis, clinical presentation and management of pancreatic diseases in pediatric IBD, including acute pancreatitis, chronic or recurrent pancreatitis, autoimmune pancreatitis and asymptomatic hyperenzymemia [12].

## 2. Methods

A comprehensive literature search was performed using the databases PUBMED MEDLINE and GOOGLE SCHOLAR, using the following keywords: [pediatric IBD] OR [children] AND [ulcerative colitis] OR [Crohn’s disease] combined with [Acute pancreatitis] OR [pancreatic manifestations] OR [autoimmune pancreatitis] OR [idiopathic pancreatitis]. We focused on the most relevant articles published in English from 2010 to May 2021, including Systematic Reviews, Consensus Guidelines, Randomized Controlled Trials, Cohort Studies and Case Reports. The selected papers were analyzed in order to extrapolate data on the etiology, pathogenesis and clinical presentation of pancreatic diseases in pediatric IBD.

## 3. Extraintestinal Manifestations of IBDs

Incidence rate of EIMs is 6–47%, and they can present during the course of the disease or seldom preceding the diagnosis of IBD [6,9,11,15,16]. Two studies addressing the incidence of EIMs in IBD have shown similar results in the pediatric setting, with respectively 28.2% and 29% of the cohorts having experienced at least one EIM during the follow-up [15,16]. For pediatric-onset UC, a recent French population-study have reported a higher frequency of EIMs both at diagnosis and during the follow-up, compared with the elderly-onset disease [17]. Interestingly, EIMs at diagnosis have been also associated with a more severe course over time [17]. Similar results have been reported for pediatric-onset CD [18], suggesting that EIMs in children may have a worse prognosis than in adults and predict a more progressive disease course [15,19,20]. Aside from the younger age, the extension of disease and the degree of intestinal inflammation also seem to be associated with the risk of development of one or more EIMs [21,22]. For instance, erythema nodosum and peripheral arthritis tend to run in parallel with the intestinal activity, while some others, such as those involving the hepatobiliary system and pancreas, typically exhibit an independent course [3].

## 4. Role of Genetics in Pediatric Pancreatitis and Its Association with IBD

A combination of genetic susceptibility, immunological disturbances and environmental factors variably participate in the pathogenesis of pancreatic disease associated with IBD [1]. Nevertheless, while the contribution of genetics to the onset and progression of pancreatitis has been widely assessed, poor are the data about its role in pancreatitis associated with IBD [23].

According to several studies on the non-IBD population, environmental factors are predominant in the case of a single episode of AP [24,25]. By contrast, cases of acute recurrent pancreatitis (ARP) and chronic pancreatitis (CP) should rise the possibility of a genetic substrate also in pediatrics [26,27,28]. Indeed, genetic causes have been proved to account for less than 10% of pediatric AP, while up to 50% of pediatric ARP and 75% of CP can be genetic based [29].

It has been proved that specific polymorphisms can act as cofactors for the disease: they may strengthen or amplify the effect of environmental triggers, contribute to a more severe course over time, increase the risk of recurrence after the first attack, impacting on fibrotic processes that may progress to a chronic disorder [25,26,27,28,29].

Most pancreatitis risk genes code for digestive proteases, trypsin inhibitor or other enzymes highly expressed in the pancreas, as well as for cytokines and chemokines involved in the inflammatory response [29,30]. The premature intra-acinar zymogens activation and the NF-kB mediated inflammatory cascade are two main pathogenetic events for the onset of AP and for the occurrence of systematic complications, so that various groups of genetic mutations may variably participate in the pathogenesis of AP (Table 1) [25].

The main genetic variants associated with premature trypsinogen activation have been identified in the Cystic Fibrosis Transmembrane Regulator (CFTR), serine protease inhibitor Kazal type I (SPINK1), Cationic Trypsinogen (PRSS1) and chymotrypsin C (CTRC) genes [29,31,32]. An impairment in SPINK1 expression has been also associated with sustained trypsin activity over time, thus leading to an increased risk of recurrence and progression of the disease [30]. Variants in SPINK1 N34S haplotype, present with a prevalence of 1–2% in the healthy population [33], have been particularly associated with a faster progression to ARP, along with an increased risk of CP by approximately 10-fold [30]. Similar evidence has been observed for CFTR, PRSS1, CTRC, and carboxypeptidase 1 gene mutations, which seem to predispose children to early-onset CP [29,34].

A recent systematic search of the literature and meta-analysis identified a significant association of SPINK1, Aldehyde Dehydrogenase 2 (ALDH2), interleukin (IL)-1B, IL-6, and IL-18 variants with the risk for AP, while variants in TNF, GSTP1 and CXCL8 were associated with disease severity [35]. Polymorphisms in the promoter regions of IL-1B, IL-6, IL-8, and IL-10 have been furthermore related to a more severe course of the disease [33].

Anyhow, in the clinical practice genetic testing is generally considered for individuals with manifested symptoms of CP or recurrent acute pancreatitis. Patients presenting with a first episode of acute pancreatitis should also be considered when they are young (<18 years), with a family history of pancreatitis or when family members are asymptomatic carriers of mutations that are known to be associated with hereditary pancreatitis (HP) [33].

Besides classic risk genes for pancreatic disease, recent studies have focused on genetic susceptibility loci shared by both pancreatitis and IBD, in order to explain the link between these two conditions.

Myosin IXB (MYO9B) gene encodes the Myosin-IXb protein, which is expressed in various tissues, including human intestinal epithelial cells [36]. It seems involved in the correct mucosal barrier function through the regulation of tight-junctions assembly and actin filament remodeling, so that specific polymorphisms of MYO9B gene have been associated with intestinal permeability impairment and conditions such as celiac disease and IBD [36,37,38,39]. Several MYO9B variants have been associated with UC, CD and pancreatitis, albeit with discordant results [40,41], and only single nucleotide polymorphism (SNP) rs1545620 has been observed in all of them [38,41].

Further studies have also highlighted that both pancreatitis and IBD share a common immune dysregulation and an altered cytokines expression, such as an abnormal elevation of IL-1β, IL-6, IL-8, and IL-10, thus supporting the susceptibility for IBD patients to develop pancreatic manifestations over time [42,43,44].

The role of MUC1 mucin for intestinal and pancreatic inflammation has been assessed in murine models as well [45]. MUC1 is a transmembrane glycoprotein normally expressed at low levels on pancreatic ductal epithelia and other epithelial cells. In mice with induced colitis, it has been shown to be abnormally expressed and hypo-glycosylated both in intestinal inflamed epithelium and in pancreas. However, a precise pathogenetic role has not been completely elucidated [45].

It is known that changes in microflora composition may result in a reduced microbial diversity, leading to immune disorders and susceptibility to disease, including pancreatitis and IBD [1,23,30]. IBD has been associated consistently with gut dysbiosis, low concentration of Firmicutes, such as Bifidobacterium, Clostridia and especially Faecalibacterium prausnitzii, and increased concentrations of Escherichia Coli [46]. Adherent-invasive E. coli are resistant to clearance by the immune system and induce an increased expression of the inflammatory cytokine tumor necrosis factor-α (TNF), which is fundamental for the onset and progression of gut inflammation [47]. Similarly, in AP and CP levels of Bifidobacterium or Lactobacillus have been shown to be lower, along with a higher concentration of Enterobacteriaceae. Such a close interaction between changes of microbiota, intestinal inflammation and pancreatic disease has been considered as the gut-pancreas axis [23]. Its pivotal role as a first line of defense against adhesive and invasive commensal bacteria has been confirmed by recently published results on glycoprotein 2 (GP2) [47]. GP2 is the most abundant pancreatic membrane protein of zymogen granule and is also expressed on microfold cells (M cells) located in the small intestine [47]. It initiates antigen-specific mucosal immune responses against bacterial invasion into intestinal epithelial cells. Indeed, it has been demonstrated that GP2-deficient colitis mice present a more severe intestinal inflammation and an enhanced E. coli population than healthy mice, proving the role of GP2 in preventing E. Coli mucosal attachment and penetration [47,48]. By contrast, an increase of GP2 expression in the pancreas has been observed in a chemically induced colitis in mice, as a consequence of the production of pro-inflammatory cytokines such as TNF. A rise in fecal GP2 concentration along with the presence of anti-GP2 antibodies has been also observed in CD patients, although the exact role of pancreatic GP2 and the pathogenetic role of anti-GP2 antibodies in IBD patients remains unclear [47,48].

## 5. Pancreatic Manifestations of IBDs

### 5.1. Acute Pancreatitis

AP consists of the activation of pancreatic enzymes inside the pancreas for various reasons, leading to a sterile inflammation of the tissue. Two large-scale studies have found an almost 4-fold and 1.5–2-fold risk of developing AP in patients with CD and UC, respectively, compared to the healthy population [6,49,50,51]. A recent meta-analysis has confirmed that IBD significantly elevates the risk of developing AP, with three times higher odds (OR, 3.11) in IBD than in the non-IBD population, and with a higher risk in CD than in UC [52]. It has been reported a significant association between AP and severe IBD, as well as with female sex [10,53]. Interestingly, based on two studies, AP in children may precede the diagnosis of IBD more frequently than in adults, so that a first episode of pancreatitis at a young age should alert clinicians to a possible underlying IBD [53,54]. Indeed, IBD is accounted among the top five causes of AP in children [55], and if other known causes of pancreatitis are not found, a not invasive work-up to exclude IBD should be warranted [9,52,53]. Anyhow, clinical presentation and course of acute pancreatitis in IBD are similar to the general population [6,56,57].

According to cohort studies on AP in IBD, an etiological classification has been proposed (Table 2) [9,52]. The first two groups include the most common causes of AP, which are not directly disease-related but rather due to side effects of medications, treatment-induced complications, or biliary lithiasis [3]. In both children and adults, drugs and gallstones represent the most common causes of pancreatitis, and for both of them the risk appears to be higher in CD patients [6,9,58]. Another cause of AP in children is represented by duodenal localization of CD with the iuxta-papillary area involvement, which may cause pancreatitis through flow obstruction, duodenal reflux into the pancreatic ducts, or rarely duodenal-pancreatic duct fistula [9,10,11]. Iatrogenic harm accompanying endoscopic procedures, such as balloon endoscopy, is considered a quite uncommon cause of AP in children, as well as primary sclerosing cholangitis (PSC) [9]. In the third group are included those conditions are included which are believed to share the same pathogenesis with IBD or to be a direct consequence of systemic inflammation and autoimmune phenomena. They are considered true EIMs of IBD (see below).

#### Diagnosis of Acute Pancreatitis

Based on established international guidelines [58,59], AP is diagnosed by at least two of the following three criteria: (i) symptoms suggestive for AP (abdominal pain, nausea, vomiting, back pain), (ii) an elevation of lipase (+/− amylase) greater than three times the upper limit of normal, (iii) and/or pancreatic inflammation on imaging.

Abdominal pain is the leading symptom of AP which can behave as possible confounder, since it may coexist in CD, UC and pancreatitis, along with other symptoms such as nausea, vomiting and diarrhea. Likewise, elevation of pancreatic enzymes is highly frequent among patients with IBD even without clinical evidence of pancreatitis, so making a proper diagnosis is not always straightforward and a relapse of IBD might be mistaken for AP and vice versa [8]. When AP is suspected, the physician should closely observe the patient and perform imaging tests in order to make diagnosis [9].

### 5.2. Drugs-Induced Pancreatitis

Molecules held responsible for AP in IBD are those commonly used in the management of the disease, such as immunomodulators (azathioprine, 6-mercaptopurine, cyclosporine), aminosalicylic acid, metronidazole and corticosteroids [10]. Azathioprine (AZA) carries the highest risk for pancreatitis, and a recent study has shown an almost six times higher chance of developing AP in children on AZA. [60]. Thiopurines-induced pancreatitis is typically dose independent and usually occurs one to three weeks after the start of therapy. There is no evidence that monitoring lipase levels can predict disease risk [6]. Thiopurines-induced pancreatitis has shown an uncomplicated and self-limiting course in most cases [61]. In UC children with clinically significant pancreatitis, current guidelines suggest discontinuing thiopurines, with subsequent enzyme levels normalization in most cases [13,60]. While thiopurine S-methyltransferase (TPMT) polymorphisms have not been associated with the risk of thiopurine-induced pancreatitis [56], it has been identified a strong association within the class II HLA region and specifically with the SNP rs2647087 that maps to the HLADQA1* 02:01-HLA-DRB1*07:01 haplotype (odds ratio, OR, 2.59) [62]. Specifically, in the study by Heap and colleagues, patients being heterozygous (A/C) and homozygous (C/C) carriers of the variant allele have shown a 2.5- and 5-fold higher risk of developing thiopurine-induced pancreatitis, respectively, compared to those who were homozygous for the common allele (A/A) [6,61,62]. A recent study on an adult IBD population has validated these findings, showing even a higher risk of pancreatitis, with 4- and 15-fold risk for rs2647087 A/C and C/C patients, respectively. In the study, an algorithm has been proposed for rs2647087 homozygous and heterozygous variant carriers, which would allow them to be excluded from AZA therapy, minimizing the risk of adverse effects (AEs), and to be treated with other more effective IBD therapies [63].

### 5.3. Idiopathic Pancreatitis

AP resulting from an unknown cause is referred to as idiopathic pancreatitis (IP). Indeed, it is worth mentioning how roughly 20–30% of pancreatitis remain of unknown origin, thus suggesting a direct pancreatic damage in IBD [10,12]. For these cases, the exact pathogenesis is still not clear, but a multifactorial mechanism is recognized. It is well known how the systemic nature of IBD tends to promote a generalized inflammatory environment and an imbalance in the coagulation state which may predispose to hypercoagulability and eventually pancreatic ischemia [61,64,65]. Moreover, in patients with active IBD, impairment in intestinal barrier function has been associated with bacterial translocation and development of infectious complications. All of the mentioned factors may therefore cooperate to the development of AP, which would furthermore explain the correlation between AP and the extent and severity of the flare-up [65]. As previously reported, some studies have focused on some genetic susceptibility loci which are shared by both pancreatitis and IBD, which may prove the link between these two conditions and make some forms of pancreatitis as true EIMs of IBD [47,48].

### 5.4. Autoimmune Pancreatitis

Autoimmune pancreatitis (AIP) is a benign fibroinflammatory pancreatic disease and a relatively newly recognized condition, particularly in children. It is characterized by a presumable dysregulation of immune tolerance toward pancreatic antigens and subsequent development of autoantibodies [66]. Specifically, a wide range of antibodies against pancreatic enzymes and other acinar antigens has been detected, including lactoferrin, carbonic anhydrase-II (CA-II), pancreatic secretory trypsin inhibitor (PST-I or SPINK1), cationic trypsinogen (PRSS1), and anionic trypsinogen (PRSS2). [67] However, none of these antibodies can be considered disease specific, especially in children, where also elevated IgG4 levels are also uncommon (22%) [67]. AIP was first reported in 1961 as a pancreatic disorder characterized by the coexistence of painless obstructive jaundice with or without pancreatic mass, hypergammaglobulinemia, autoantibodies and a prompt response to corticosteroids [68]. According to the different histologic patterns, two well-differentiated types of AIP are recognized in adults: (i) type 1, or lymphoplasmacytic sclerosing pancreatitis (LPSP), involving lymphocytes with infiltration by IgG4-positive plasma cells, and (ii) type 2, or idiopathic duct-centric pancreatitis (IDCP), showing neutrophilic infiltration with granulocytic epithelial lesions (GELs) (Table 3) [69,70]. Type 1 AIP usually occurs in older people and is currently considered a pancreatic manifestation of IgG4-related disease [71]. Type 2 AIP is not characterized by increased IgG4 levels and affects mostly young adults. An association between AIP and IBD has been documented in the past, and in recent years AIP is being increasingly diagnosed both in UC and CD, despite the fact that a proper diagnosis can be challenging [72,73,74,75]. Nearly 50 cases of AIP have been diagnosed in children so far [75,76,77,78], with jaundice and abdominal pain representing the most common symptoms of presentation. Further characteristics of the pediatric cases were a median age at diagnosis of 13 years old (range 2–17), male predominance, an optimal response to steroids and a low rate of serum IgG4 elevation [78]. Pediatric AIP can be associated to IBD in up to 25–27% of cases and usually manifests in the form of UC, with the majority of cases represented by type 2 AIP [14,79]. Despite the absence of clear and established diagnostic criteria for its diagnosis [80], data collected suggest that pediatric AIP represents a distinct subtype of pancreatitis that may express histopathological features of both types [65,81,82], including: (i) higher rate of abdominal pain and/or obstructive jaundice at diagnosis, (ii) low rate of high IgG4 levels, (iii) ductal or parenchymal abnormalities on imaging, (iv) lymphoplasmacytic, granulocytic infiltrate with fibrosis, and (v) prompt response to steroid treatment.

Recently, the first pediatric AIP recommendation statements have been developed by the “International Study group of Pediatric Pancreatitis: In search for a cure” (INSPPIRE) in order to standardize diagnosis and treatment [83].

As for diagnosis of AP, transabdominal ultrasound is suggested as first line tool to diagnose pediatric AIP, showing the inflammatory process as a sausage-shaped pancreas with an irregular narrowing of the pancreatic duct [84]. Nevertheless, magnetic resonance cholangiopancreatography (MRCP) may be required in order to identify more specific pediatric AIP findings (i.e., capsule-like rim, main pancreatic duct irregularity, common bile duct tapering within an enlarged pancreatic head) [83].

Corticosteroids (oral prednisone, 1–1.5 mg/kg/day to a maximum of 40–60 mg given in one or two divided daily doses for two–four weeks) are recommended as the first line therapy in children. However, a watchful waiting approach may be considered, given the possibility of a self-resolution of the disease and the risk of side effects of steroids [83]. In case of highly active or refractory AIP, other therapies that have been reported to be effective are represented by high-dose methylprednisolone (two courses at a dose of 500 mg for three days), thiopurines (AZA or 6-mercaptopurine), mycophenolate mofetil, or infliximab (above all in patients with concomitant IBD) [67].

### 5.5. Asymptomatic Elevation of Pancreatic Enzymes

The increased level of serum pancreatic enzymes (amylase and/or lipase) in the absence of abdominal pain and pancreatic involvement at imaging tests is defined as “benign pancreatic hyperenzymaemia” [12]. It has been observed in up to 14% of asymptomatic IBD patients (>2 times the upper limit of the normal for amylase and lipase), being not related to other causes [56]. Despite some studies have not found an association between the degree of enzymes elevation and activity of IBD [8], in some others increased amylase/lipase levels were correlated with the extent and severity of CD [53,85]. Currently, the underlying mechanism for asymptomatic elevation of pancreatic enzymes in IBD has not been elucidated, but suggested etiologies include immune abnormalities, microcirculation disorders, malnutrition, dehydration, enterobacteria with the ability to produce amylase, and the abnormal absorption of pancreatic amylase/lipase from the inflamed gut into the bloodstream [9].

In case of an IBD patient with persistent hyperenzymaemia, it is mandatory to screen him for other possible causes, including salivary gland disease, macroamylasaemia, renal impairment and familiar pancreatic hyperenzymaemia [56]. Among them, pancreatic diseases should always be ruled out, since roughly 25% of patients presenting increased pancreatic enzyme levels at the onset tends to develop AP in the following six months [8,53,65]. Anyhow, its clinical significance over time remains undefined, and even in those developing AP, prognosis turned out to be satisfactory in most cases [65].

## 6. Discussion and Conclusions

**Figure 1:**
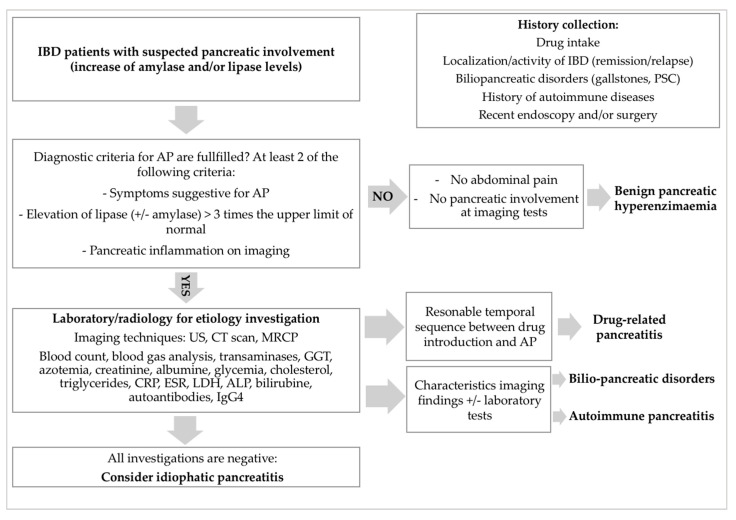
Diagnostic approach for suspected pancreatic disease in IBD pediatric patients.

Among the broad spectrum of EIMs of IBD, pancreatic disorders are not uncommon and are sometimes underestimated [8]. However, increasing knowledge of their pathophysiology and clinical characteristics has led to a better approach to these conditions (Figure 1).

AP is the most frequent pancreatic manifestation in IBD and in some cases it can even precede the onset of the intestinal disease [53,54]. For this reason, a first episode of pancreatitis, especially at a young age and without other known causes should alert clinicians to a possible underlying IBD and lead to a not invasive work-up to exclude the intestinal disease [52,53]. According to both adult and pediatric cohort’s studies, the most common causes of AP are not directly IBD-related, but rather the consequence of toxicity of drugs used for the treatment of IBD or due to a concomitant biliary lithiasis [9,52]. Thiopurines-induced AP has shown to usually occur after the first three weeks of treatment and to have an uncomplicated and self-limiting course after withdrawal of the drug in most cases [6,60].

However, regardless of whether it is drug- induced or not, the management of AP in IBD patients should follow the same protocols as in the general population, based on fluid therapy, electrolyte replacement, pain control, and nutritional support since these cases of pancreatitis have shown mostly a mild course [58,59].

Some AP forms, where the diagnostic work-up is normal and no etiology is found, so “idiopathic” by definition, can be considered as true EIMs of IBD and a direct consequence of the systemic inflammation. These forms tend to run in parallel with the intestinal inflammation and the disease flares, so that controlling the IBD activity may produce an improvement in the concomitant AP [10,65].

AIP is a rare and newly recognized condition in children, since most cases have been described in adults so far. It has been described as being associated with other autoimmune disorders, including UC, CD and sclerosing cholangitis, in up to 27% of patients. In a recent retrospective study, pediatric AIP has shown a distinct presentation from adults with features similar to type 2 AIP [14]. Abdominal pain, weight loss, and fatigue were the most consistently reported symptoms in children. Associated features were a low rate of IgG4 elevation, a combination of lymphoplasmacytic inflammation, pancreatic fibrosis, and ductal granulocyte infiltration at the histopathological examination, hypointense global or focal gland enlargement, main pancreatic duct irregularity, and common bile duct stricture at the imaging studies [14]. Based on these findings, the diagnosis of pediatric AIP can be made based on the combination of clinical presentation and radiological evaluation, possibly complemented by histopathological findings [81]. Due to the possibility of other autoimmune diseases, including IBDs, a multi-disciplinary approach is needed to exclude comorbidities. Most patients presented a good short-term clinical response to corticosteroids (27/29, 93%), defined as clinical symptom resolution, with the administration of oral prednisone, 1–1.5 mg/kg/day to a maximum of 40–60 mg given in one or two divided daily doses for two to four weeks [14]. Interestingly, a minority of patients had disease resolution without any treatment, so that a watchful waiting approach may be taken into account [14].

Despite the available recommendations, the broad spectrum of pancreatic manifestations in pediatric IBD may still represent a challenge for several reasons. The leading symptom of AP is abdominal pain, so that pancreatitis often mimic a relapse of intestinal disease. By contrast, the elevation of pancreatic enzymes is highly frequent among patients with IBD, even without clinical evidence of pancreatic disease [8]. Pathogenesis and prognosis of asymptomatic enzymes elevation in IBD patients are yet to be explored but it has been associated with the subsequent development of pancreatic disease over time in some cases [53,65]. For this reason, considering that these two conditions may overlap and be difficult to differentiate, an appropriate diagnostic workup such as the regular measurement of serum lipase and amylase levels should be warranted, since the proper recognition and early management of both IBD and AP are crucially important.

## Figures and Tables

**Table 1:** Genetic susceptibility of acute pancreatitis (based on [25]).

Stage of AP	Pathological Event	Susceptibility Genes
Early Stage	Premature trypsinogen activation	PRSS1, SPINK1, CTRC
	NF-kB activation	Interleukin genes, antioxidant enzyme genes, ACE genes, MIF, iNOS, COX-2, MYO9B
Late Stage	Severity	TLR genes, CD14, MCP-1, HBD genes, MBL2
	Complications	TNF-a genes, IL-10, TLR4

AP, acute pancreatitis; PRSS1, cationic trypsinogen; SPINK1, serine protease inhibitor Kazal type 1; CTRC, chymotrypsin C; ACE, angiotensin-converting enzyme; MIF, migration inhibitory factor; iNOS, inducible nitric oxide synthase; COX-2, cyclooxygenases 2; MYO9B, myosin IXB; TLRs, toll-like receptors; MCP-1, monocyte chemoattractant protein-1; HBDs, human β-defensin 2; MBL2, mannose-binding lectin 2; TNF-α, tumor necrosis factor-α; IL-10, interleukin 10; TLR4, toll-like receptor 4.

**Table 2:** Etiology of acute pancreatitis in IBD (based on [9,10,52]).

1. Treatment-induced: - Drugs: Immunomodulators (azathioprine, 6-mercaptopurine, cyclosporine) Salicylates (sulfasalazine and 5-aminosalicylic acid) Antibiotics (metronidazole) Corticosteroids (prednisone, budesonide) Post-endoscopic retrograde cholangiopancreatography - Post-enteroscopy
2. “Classical” risk factors: - Biliary obstruction (gallstones) - Ethanol
3. Disease-related: - Autoimmune pancreatitis - Idiopathic pancreatitis - Duodenal Crohn’s disease - Primary sclerotizing cholangitis associated pancreatitis

**Table 3:** Differential diagnosis of Type 1 and Type 2 AIP (adapted from [13,14,60]).

	Type 1 AIP	Type 2 AIP	Pediatric AIP
Age at diagnosis (years)	Adults (60s)	Adults (40s), adolescents	13 (range 2–17)
Sex	M > F	Equal distribution	M > F
Symptoms	Painless obstructive jaundice, with or without a pancreatic mass		Abdominal pain, jaundice, weight loss, fatigue
Serum IgG4 elevation	>90% of cases	¼ of cases	Uncommon
Histology	Lymphoplasmacytic sclerosing pancreatitis with “storiform” fibrosis, obliterative phlebitis and IgG4+ plasma cells (>10 per high-power microscope field)	Idiopathic duct-centric pancreatitis with granulocytic epithelial lesions (GELs)(with or without lobular neutrophil infiltration)	Similar to Type 2 AIP
Relapse rate	High (24–52%)	Low (0–27%)	Unknown
Extra-pancreatic manifestations described	IgG4-related disease, Sclerosing cholangitis, Sialoadenitis, Retroperitoneal fibrosis, Interstitional nephritis	IBD in ≥30% of cases (mostly UC)	CD, UC, autoimmune glomerulonephritis, autoimmune hemolytic anemia
Treatment in children	Oral prednisone, 1–1.5 mg/kg/day to a maximum of 40–60 mg given in 1 or 2 divided daily doses for 2–4 weeks. A watchful waiting approach may be considered.		
Response to steroids	Usually rapid (<2 weeks)	Extremely rapid	Usually rapid

AIP, autoimmune pancreatitis; UC, Ulcerative Colitis; CD, Crohn’s Disease.

## Data Availability

The data presented in this study are available on request from the corresponding author.

## Abbreviations

AP: acute pancreatitis; IBD: inflammatory bowel disease; CT: computed tomography; IgG4: immunoglobulin G4; PSC: primary sclerosing cholangitis; ERCP: endoscopic retrograde cholangiopancreatography; MRCP: magnetic resonance cholangiography; GGT: γ-glutamyl transferase; CRP: C reactive protein; ESR: erythrocyte sedimentation rate; LDH: lactate dehydrogenase; ALP: alkaline phosphatase.
